# Controlling Photoinduced Electron Transfer Via *Defects* Self-Organization for Novel Functional Macromolecular Systems

**DOI:** 10.2174/1389203715666140327104023

**Published:** 2014-06

**Authors:** Gaetano Campi, Gabriele Ciasca, Nicola Poccia, Alessandro Ricci, Michela Fratini, Antonio Bianconi

**Affiliations:** 1 Institute of Crystallography, CNR, Via Salaria Km 29.300, 00015, Monterotondo Roma, Italy;; 2 Institute of Physics, Catholic University, 00168 Rome, Italy;; 3 MESA+Institute for Nanotechnology, University of Twente, P. O. Box 217, 7500AE Enschede, Netherlands;; 4 Deutsches Elektronen-Synchrotron DESY, Notkestraße 85, D-22607 Hamburg, Germany;; 5 Fermi Center, Piazzale del Viminale, 00187 Roma, Italy;; 6 RICMASS Rome International Center for Materials Science Superstripes, via dei Sabelli 119A, 00185 Roma, Italy

**Keywords:** Charge separation, electron transfer, lab on chip, photo-induced phase transition, synchrotron radiation, X-ray micro-diffraction.

## Abstract

The electrons transfer (ET) from an atom or a molecule, *donor* (D), to another, *acceptor* (A) is the basis of many fundamental chemical and physical processes. The ET mechanism is controlled by spatial arrangements of *donor* and *acceptors: *it’s the particular spatial arrangement and thus the particular distance and the orientation between the electron donors and acceptors that controls the efficiency in charge separation processes in nature. Here, we stress the importance of this concept reviewing how spatial distribution of atomic and molecular self-assembly can determine the quality and physical features of ET process from biology to material science. In this context, we propose novel *lab-on-*chip techniques to be used to control spatial distribution of molecules at nanoscale. Synchrotron source brightness jointly to focusing optics fabrication allows one nowadays to monitor and visualize structures with sub-micrometric spatial resolution. This can give us a new powerful tool to set up sophisticated X-ray imaging techniques as well as spectroscopic elemental and chemical mapping to investigate the structure-function relationship controlling the spatial arrangement of the molecules at nanoscale. Finally, we report intriguing recent case studies on the possibility to manipulate and control this spatial distribution and material functionality at nanoscale by using X ray illumination.

## INTRODUCTION

In living systems the directional transport of electrons (ET) controlled by spatial arrangements of *donors* (D) and *acceptors *(A)* is a fundamental process *for charge separation to convert light energy into chemical energy. In photosynthesis light illumination produces electron-hole excitations followed by charge separation controlled by the reaction centers in the various membrane complexes. One of the most intriguing problems of electron transport in biological systems is how an electron can be moved over long distances about 10-30 nm with little loss of energy [1]. Early theories, in the beginning of the 1940’s, were based on electronics jumps from enzyme to enzyme immobilized in membranes by using energy bands analogous to those found in semiconductors [2]. Later, various dynamical models proposed protein conformational changes pushing electrons [3, 4]. The view changed dramatically in the 1960’s when De Vault and Chance demonstrated that a cytochrome in the photosynthetic bacterium *Chromatium vinosum* was oxidized with a half-time of 2 μs [5]. Furthermore, the same author found that the time constant for this electron transfer reached a limiting value of 2 ms at 100 K, remaining constant down to4.5 K [6]. At the beginning of the 1990’s, the ET was found to occur in few ps in purple bacteria at room temperature [7, 8]. 

Between various mechanisms proposed for ET explanation, the treatment of the reactant-product transition probability produces the semiclassical Marcus equation [9] for the rate, *k_ET_*, of non-adiabatic electron transfer (ET) between a donor (D) and acceptor (A) held at fixed distance:

(1) $$k_{ET}=\sqrt{\frac{4\pi^{3}}{h^{2}\lambda K_{B}T}}H_{AD}\exp\left[-\frac{\Delta G+\lambda }{4\lambda K_{B}T}\right]$$

Here ΔG is the change in free energy associated with the ET reaction; λ is reorganization arising from *i*) the nuclear deformation of the reactants, *ii*) the intra-molecular reorganization energy, and *iii*) the solvent reorientation. H_AD_ represents the electronic coupling between donor and acceptor states defined as

(2) $$H_{AD}=<\Psi_{A}\left|H\right|\Psi_{D}>$$

where Ψ_A_ and Ψ_D_ are the acceptor and donor electronic wavefunctions, respectively, and H is the Born-Oppenheimer (rigid nuclei) electronic Hamiltonian for the system. The factors λ and ΔG depends on the properties of the solvent, the donor, the acceptor and their mutual distance. The reaction is called *adiabatic* or *non-adiabatic* depending on the H_AD_ value.

Classical ET theory is well suited to treat ET reactions in the adiabatic limit where H_AD_<KT. As the electronic coupling increases, one falls in the so-called ‘strong coupling’ limit and a quantum-coherent treatment enters the scene [10]. In the strong coupling limit the donor and acceptor electronic states mix to produce new delocalized states (Frenkel excitons). These states, are slightly perturbed by the interactions with the solvent and the energy is quantum shared among several A-D instantaneously, giving rise to the so-called *wave-like transfer*. From the experimental point of view, the development of two-dimensional electronic spectroscopy (2DES), has been able to experimentally detect signatures of coherence dynamics. Indeed, to date, 2DES, and in particular two dimensional photon echo (2DPE), is the most common spectroscopy used to explore quantum effects [11, 12]. 

Although experimental evidence is accumulating, the quantum effects in ET occurring in biological systems remain highly debated. In any case, despite the various theories and the experimental findings, the possibility to mimic the natural process is strongly correlated to spatial chromophores arrangement [13-15]: it’s the unique spatial arrangement of the structural constraints controlling both the distance and the orientation between the electron donors and acceptors that produces the high efficiency light-charge separation processes. This issue inspired scientists to engineer artificial systems that reproduce the conversion of light into electrical energy. 

## FROM PROTEINS TO D-B-A ARTIFICIAL COMPOUNDS

ET in living systems involves proteins such as flavodoxins, blue-copper proteins, iron-sulfur proteins, and cytochromes. [16, 17] In these proteins ideal metal ions sites would provide very fast electron transfer if the metal coordination spheres could come into contact with each other (i.e., as in small-molecule outer sphere reactions, in which the unimolecular rate constant for electron transfer would be close to 10^10 ^s^-1^ if there were no thermodynamic barriers). Lewis *et al.* showed that ET in DNA depends on both the distance between the electron donor and acceptor and the nature of the molecular structure separating the donor and acceptor [18]. They showed that the distance decay of ET in DNA rates varied with the energetics of the donor and acceptor relative to the bridging molecules, B.

Inspired by these complex protein systems, many studies have been devoted to designing simpler artificial light harvesting architectures called D–B–A compounds, based on an electron-donating moiety D and an electron-accepting moiety A covalently bound to the ends of some rigid bridging structure B. Several donor–bridge–acceptor (D–B–A) systems have been synthetized; interesting bridges are represented, for examples, by self-assembled monolayers of -sulfur functionalized cyclohexylidenes [19].

Using the semi-classical theory (eq. 1), it is possible to define an effective tunneling barriers ΔE_eff_ in terms of the exponential decay constant, (, describing the variation of rates with distance

(3) $$DE_{eff}=\;0.952\;eV\;{Å}^{2}b^{2}$$

This tunneling depends on the properties of the D–B–A compound and the surrounding solvent. In many cases, especially in the non-adiabatic limit where the mutual distance is large, the electronic properties of the bridge are a major factor in determining the rate of ET [20]. Thus, it is just the incorporation of bridge chromophores (B) with its spatial arrangement between A and D that improves energy transfer towards acceptors (A), recalling the doping mechanism for semiconductors.

## D-A CRYSTALLINE LAYERS WITH *ATOMIC-DEFECTS*

Simpler systems for photoinduced ET are constituted by crystals, where ET is promoted by outer sphere (OS) electrons jumping to the *conduction band* generated by a periodic arrangement of electron density. Crystals can be viewed as the simplest systems in nature due to their long range ordered periodic structure. Indeed, their homogeneous structure has been taken as a basic frame for the solid-state theories development. Anyway, in the last decades homogeneity and long range order has been broken also in these idealized simple systems. It was due to the presence of atomic defects. Wagner and Schottky [21] showed through statistical thermodynamic treatments of mixed phases that crystal structures are not ideal. Some lattice sites can be empty (vacant) and extra atoms may occupy the interstitial space between the atoms on the lattice sites. The empty lattice sites are termed *vacancies* and the extra atoms *interstitial atoms*. Following Wagner and Schottky, all crystalline solids contain vacancies and extra atoms and exhibit deviations from the ideal structure at any temperature. 

These defects are at the base of many of their properties, including their ET process. It’s well known how in semiconductor devices field defects push more electrons in the conduction bands. Beyond semiconductors, defects play a fundamental role in superconductivity [22-24], where their concentration is able to switch a material from insulating to Fermi liquid metal. In Fig. (**1**) we draw a scheme for ET in cuprates, a well-known class of high T_c_ superconductors: stacks of atomic layers playing the role of Acceptors to storage electrons and Donors to storage holes. Electron and holes defects can be introduced by doping or, as we will see ahead, by illuminating the sample.

What appeared quite surprising in recent researches is that not only the defects content but also the 3D defects spatial distribution significantly affects the transport properties [25-28]. Thus, spatial distribution of acceptor, donors and bridges can be considered a key point to be addressed in order to get deeper insight on ET in nature, ranging from biological to material science field.

## CONTROLLING AND VISUALIZING SELF-ASSEMBLY AT NANOSCALE

Self-assembly provides a facile means for organizing molecules into supramolecular structures that can bridge length scales from macroscopic down to nanometers dimensions. These structures can provide charge conduits that can efficiently drive electrons and holes between reaction centers. Small and wide angle X-ray scattering (SAXS/WAXS) from non-covalent aggregates using a synchrotron source is a powerful tool for the elucidation of their solution phase structures and formation kinetics [29-32]. New computational approaches allow SAXS/WAXS data to be interpreted

in terms of coordinate models and molecular dynamics simulations and can be used to refine the structures of self-assembled systems [33-35]. However, self-assembly does not allow a full control of the molecular spatial arrangement; this constitutes a crucial point in structure-function relationship since understanding interactions between A and D block-units, differently arranged in space, can be critically important [36-39]. 

**Fig. (2):**
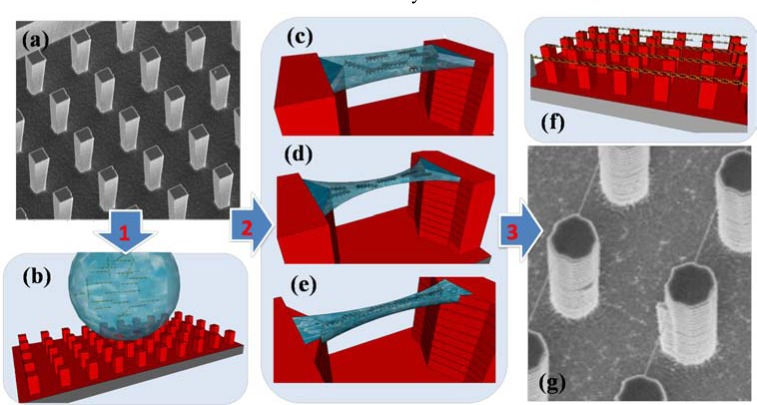
Schematic flow chart describing a novel method to realize highly ordered arrays of stretched and suspended DNA strands by using superhydrophobic surface. **Panel a** shows a typical superhydrophobic patterned surface fabricated by electron beam lithography on silicon substrate. The peculiar device morphology, made of high aspect ratio pillars, hinders the surface wettability forcing the water to assume very high contact angle (>150°). The operation principles of the above mentioned device is summarized in **panel b-f**. A few microliters water droplet containing DNA is deposited on the superhydrophobic surface (**panel b**), specifically designed to obtain a fine control of the droplet dewetting dynamics. To this purpose, silicon pillars entail tips (hundreds of nanometers in size) which, under evaporation conditions, favor the formation of water capillary between adjacent pillars. A schematic view of these nanostructures is shown in **panel c**, **d** and **e**. The water capillary evaporation pushes together the DNA strands (**panel d**) and allows the formation of stable DNA bundle precisely positioned on the tips side (**panel a**). The above described device structure allows to obtain highly ordered arrays of strictly oriented DNA bundles, as schematically shown in **panel f**. **Panel g** shows a typical SEM image of a couple of ordered arrays of DNA filaments stretched and suspended above superhydrophobic silicon pillars. As can be clearly noticed, DNA bundles appears to be precisely positioned on the same pillar position as a consequence of the above described mechanism.

In this context it was proposed to use nanotechnology and sample manipulation by external stimuli to reach a better control of atomic and molecular spatial arrangement. In particular, the possibility offered by new nanotechnology to design devices able to control spatial arrangement and concentration of molecules could play a crucial role [40]; for example, the development of novel techniques based on droplets deposition on superhydrophobic surfaces (SHSs) can be used to study chemical reactions, interactions, aggregation/precipitation processes and phase transitions by in situ X-ray scattering, diffraction and spectroscopy [40, 41]. Such studies took also advantage of drop evaporation allowing measurements in a dynamical concentration status. Very recently, an *ad hoc* designed superhydrophobic surface has been used to realize large ordered arrays of strictly oriented DNA filaments, as shown in (Fig. **2**). This highly order and controlled configuration could contribute to boost the fundamental research on electron transfer in biological systems.

**Fig. (3):**
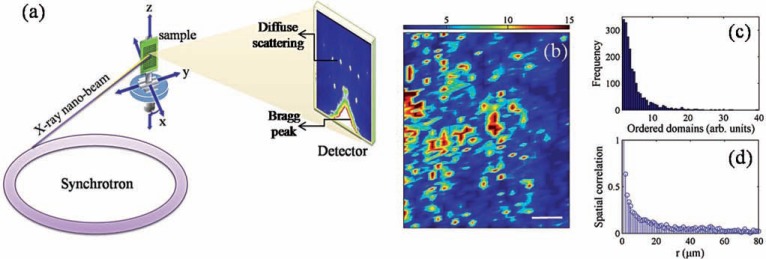
(**Panel a**) Typical experimental setup for high spatial resolved porobes. Synchrotron X ray in focused on (sub)micrometric sample areas. A single collected frame, onto an area CCD detector, allows us to get information about average crystalline structure (Bragg peak) and about defects organization (diffuse scattering). High precision mechanical x-y-z stages allow the sample to move under the beam. (**Panel b**) Mapping of intensity measured from diffuse scattering due to satellite reflections associated to interstitial oxygen defects. The bar is 20 microns. Both the (**c**) spatial distribution and the (**d**) spatial correlation of satellite intensity, related to the density of ordered domains, follow a clear power-law behavior, indicating a scale-free, fractal structural organization of ordered interstitials oxygen. Details about spatial distribution and spatial correlation calculations can be found in ref. [25]. Is this particular defects topology that gives the optimum superconducting temperature.

Improvements in synchrotron source brightness and focusing optics fabrication, make X-ray imaging techniques at nanoscale actually available, resulting in high-impact applications in several fields ranging from material science to bio-medicine. Thus, the application of spectroscopic elemental and chemical mapping, microdiffraction-based structural analysis, and coherent methods for nanomaterials imaging on the structures deposited on the described (SHSs) nanodevices could allow to investigate the structure-function relationship controlling the spatial arrangement of the molecules. In Fig. (**3**), we illustrate the case of nanodiffraction on heterogeneous crystals. The high brilliance of synchrotron radiation, in combination with active X-ray focusing optics, permits one to hit small sub-micrometric zones with high photon flux. In this way we can *i*) visualize weak, but important features in structural properties of a material, thanks to the high flux; *ii*) map these features with submicrometric spatial resolution thanks to the nano beams provided by the focusing optics. Fig. (**3a**) shows the scheme of a typical experimental setup permitting the detection of weak diffuse scattering as satellite reflections surrounding strong Bragg peak. While Bragg peaks account for average long-range ordered crystalline structure, diffuse scattering gives information on local scale or short-range ordered structures. In the specific case, satellites represent defects ordering in a La_2_CuO_4+y_ crystal. In Fig. (**3b**) we show the mapping of the ordered defects domains on the sample. Basic spatial statistics analysis (Fig. **3C**, **3d**) show that the defects arrange themselves in a scale free network configuration. Even more remarkably, is the fact that different spatial arrangement of the same defects content, gives rise to different properties, such as different superconducting critical temperatures [25].

## PHOTO INDUCED CHARGE ORDERING: X-RAY DIFFRACTION PUMP AND PROBE 

As mentioned before, external stimuli such as thermal treatment, doping, B incorporation, photo-illumination can help in controlling structure and functionality of material. Let’s consider here defects self-assembly controlled and monitored under photo-illumination. Kawashima measured large persistent photoconductivity in oxygen-reduced YBCO/LCMO superlattices grown by pulsed laser deposition [42] while Peña *et al.* reported a large *transient *photo-induced enhancement of the superconducting critical temperature in epitaxial YBa_2_Cu_3_O_6.7_*/*La_0.7_Ca_0.3_MnO_3_ bilayers upon visible light illumination [43]. Recently, Fausti *et al.* used mid-infrared femtosecond pulses to transform the stripe-ordered compound, not superconducting La_1.675_Eu_0.2 _Sr_0.125_CuO_4_, into a transient three-dimensional superconductor [44, 45]. 

**Fig. (4):**
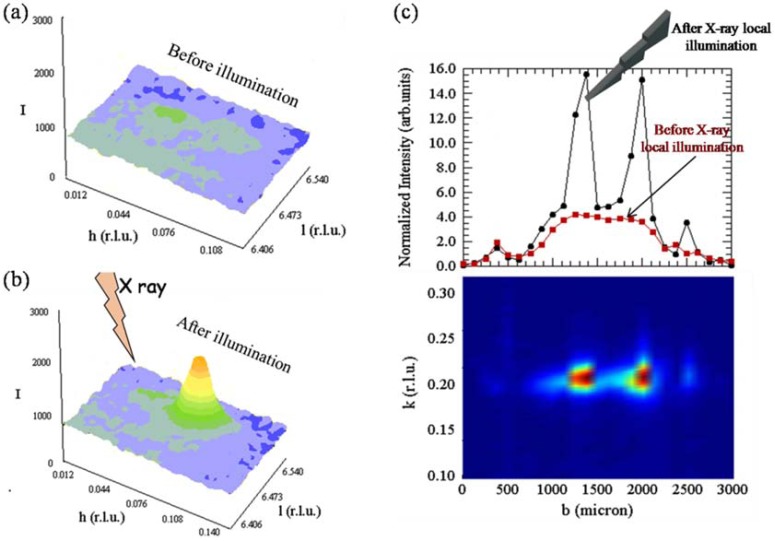
(**Panels a-b**) Photo-induced interstitial oxygen ordering in in La_2_CuO_4+y_. (**Panel c**) The defects ordering, produced by X ray illumination, is obtained locally, illuminating the sample in three different points. The difference with the satellite intensity before the illumination (red squares), measured along the b crystallographic direction of the sample, is well appreciable in the three illuminated points.

Besides optical wavelengths, also different energies (e.g., X-rays) produce intriguing morphological as well as functional effects on materials. In this framework, a new *pump and probe* experimental approach, based on X-ray diffraction performed with synchrotron radiation has been recently proposed. In this approach the X-ray photon beam works at the same time as pump and as probe on the portion of sample illuminated. Here we’ll show some significant results in La_2_CuO_4+y_ in order to offer an intriguing aspect in field of photo induced charge separation. La_2_CuO_4+y_ is a relatively simple high-*T*_c_ superconducting system, with the negatively charged CuO_2_ layers intercalated by rock salt La_2_O_2 _spacer layers. Above 370 K the doping oxygen interstitials, y, releasing holes in the basal CuO_2_ planes, become disordered; if a sample is heated above this temperature and then quenched to low temperatures it has a poor superconducting order. However, by illuminating such a disordered sample with X-rays at room temperature, it was observed nucleation and growth of ordered domains (see Fig. **4A**), giving a recovery of a robust high-*T*_c_ state. It is now understood that it is a particularly ordered spatial distribution of oxygen interstitials that hosts the highest temperature superconductivity: more specifically, the optimal superconductivity occurs when defective ordered domains percolates into a fractal network [25, 26]. It is possible to construct such network using (sub)micro-beams as a pen, in order to design arbitrary patterns of higher superconducting paths.

## Figures and Tables

**Fig. (1):**
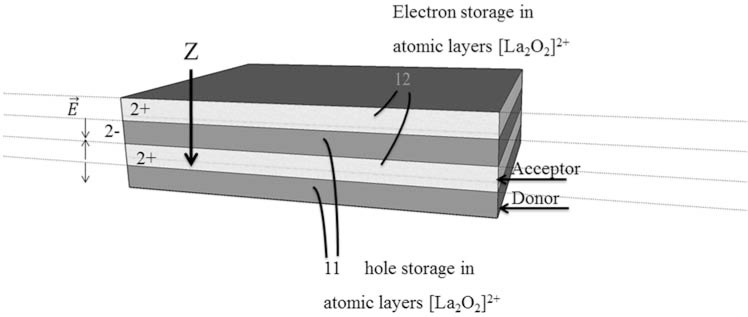
Architecture of a typical high temperature superconductor made of stacks of atomic layers playing the role of Acceptors to storage photo-induced electrons and Donors to storage photo-induced holes.

## SUMMARY

Electron transfer mechanism is a long-standing puzzle tackled by a common effort from different research fields. Huge advances have been made from the first attempts in the explanation of the phenomenon, leading to new modern theories based on quantum coherence and advanced experimental techniques based on ultra-fast spectroscopy. Although the remarkable advances, people are not yet able to mimic natural ET processes, meaning that new paths could be followed. In this review, taking inspiration from the new results in “*simpler*” materials, we underline the importance of spatial distribution of A-B-D systems for the material functionality. The high performance of efficiency for photosynthetic networks found in nature is related to the spatial distribution of various chromophores transporting energy to reaction centers. Thus, the ability to study several spatial arrangements of the chromophores, could allow us to correlate structure to efficiency. For this aim we propose to use novel lab-on chip techniques for manipulating and controlling this spatial distribution, jointly to advanced structural probes based on bright sources and focusing optics for monitoring and visualization.
